# Carcinome à cellules de Merkel: Une nouvelle observation

**DOI:** 10.11604/pamj.2015.22.256.8202

**Published:** 2015-11-18

**Authors:** Ramli Inssaf, Hassam Badredine

**Affiliations:** 1 Service de Dermatologie et Vénérologie, CHU Ibn Sina, Université Mohammed V, Rabat, Maroc

**Keywords:** Carcinome de Merkel, nodule, tumeur neuroendocrin, Merkel cell carcinoma, nodule, neuroendocrine tumor

## Image en medicine

Le carcinome à cellules de Merkel (CCM) est une tumeur neuroendocrine très rare, de localisation cutanée primitive et d’évolution très grave. Sur le plan clinique, il s'agit d'un nodule inflammatoire dermo-hypodermique dur, bien circonscrit, dont la taille moyenne est de 1 à 3 cm, localisé sur les membres ou le visage. Des études récentes ont permis d'isoler un nouveau virus étroitement associé au CCM, dénommé MCPyV. Ce virus, appartenant à la famille des polymavirus, a suscité un regain d'intérêt, tant pour le suivi et la prise en charge médicale que du point de vue cognitif. Le traitement du CCM est mal défini. La chirurgie reste le principal traitementaux stades localisés (stade I et II). La radiothérapie adjudante augmente de façon significative le taux de survie à deux ans. Dans les formes métastatiques, la chimiothérapie peut être utilisée comme un traitement palliatif. Par ailleurs, les thérapies ciblées restent l'espoir dans ces formes avancées. Nous rapportons le cas d'un homme de 93 ans, consultait pour un nodule angiomateux ferme de l'avant bras gauche (A) dont l’étude immuno-histologique a permis de retenir le diagnostic d'un CCM (B). Une chimiothérapie palliative à base de Cisplatin était indiquée. Le patient est décédé 8 mois après le diagnostic du CCM.

**Figure 1:**
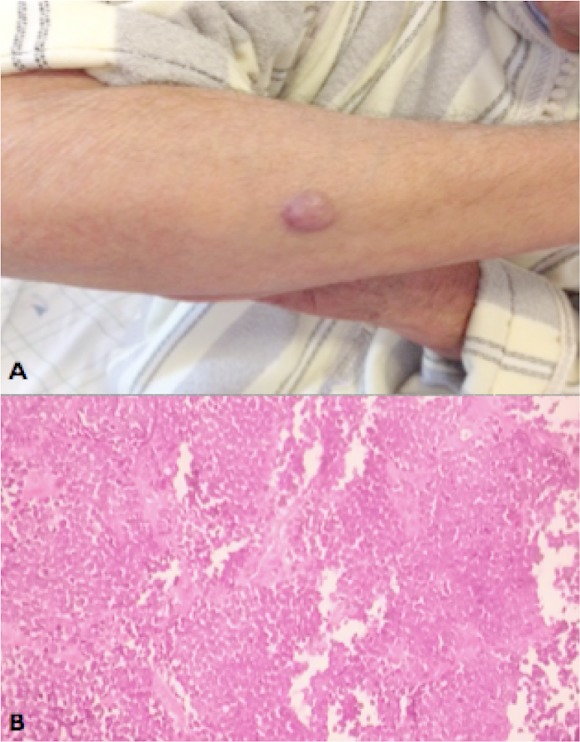
A) nodule ferme érythémato-violacé de la face externe de l'avant bras; B) prolifération tumorale massive monomorphe à activité mitotique élevée

